# Social media use, and fear of COVID-19 among Ghanaian university students: the moderating role of gender

**DOI:** 10.1186/s40359-022-00915-4

**Published:** 2022-08-29

**Authors:** Esther K. Malm, Mabel Oti-Boadi, Nutifafa Eugene Yaw Dey, Abigail Esinam Adade, Godwin Ocansey

**Affiliations:** 1 Department of Psychology, Murray State University, Murray, KY USA; 2 Department of Psychology, University of Ghana, Legon, Accra, Ghana

**Keywords:** Gender, Social media use, Fear of COVID-19, University students, Ghana

## Abstract

**Background:**

COVID-19 and its associated social restrictive measures and lockdowns exacerbated the use of social media and other technological facilities for communication. This study, therefore, examined Ghanaian students’ social media use and its relationship with fear of COVID-19, paying close attention to the moderating role of gender.

**Methods:**

A correlational online survey was used to collect data from a purposive sample of 209 University students in June and July 2020. Participants completed online measures on social media use and fear of COVID-19. Statistical analyses including independent-t test, Pearson correlation test and moderation analysis in PROCESS were conducted using SPSS v.24.

**Results:**

Findings revealed that the mean scores of social media use and fear of COVID-19 did not statistically differ by gender. However, social media use had a small and positive association with fear of COVID-19 (*r* = 0.18, *p* = 0.009). Furthermore, gender was a significant moderator of the relationship between social media use and fear of COVID-19. Specifically, the increased use of social media resulted in greater experiences of fearing COVID-19 for females (*B* = − 0.24, *p* = 0.034) compared to males.

**Conclusion:**

Although social media was useful in connecting with people and accessing pandemic-related information, our findings clearly suggest that overuse or over-engagement with social media was problematic, especially for females. Aside from developing interventions to reduce students’ fears of COVID-19, appropriate usage of social media should be advocated.

## Introduction

The novel coronavirus (SARS-CoV-2), popularly known as COVID-19, is a highly infectious virus discovered in China, Wuhan in the later part of 2019. It was highly transmissible across the globe and was officially declared a pandemic by the World Health Organization on 11th March 2020 [1]. Research indicates that COVID-19 spreads rapidly through physical and social contact with others who may have contracted the disease [2]. In Ghana, the first two cases were identified in March 2020 with and was reported to have quickly spread in communities [3]. The cases increased quickly to 54,771 people as of 31st December 2020, making Ghana the country with the second highest records in West and Central Africa [4]. To control the spread, there were restrictions instituted by the government of the day which included an immediate ban on all public gatherings, and mandates for frequent hand washing, use of masks (with punitive consequences), and social distancing [3]. Such stringent measures suddenly stirred panic and uncertainty among Ghanaian particularly because such a ban for social interaction had never occurred before in a country with a culture deeply rooted in social interactions, particularly on weekends. The restrictions also caused fury, panic buying and changes of lifestyles [5]. It also stoked many social media discussions of both inaccurate and accurate information, as well as the consequent fears experienced globally [6, 7].

Around the world, COVID-19 has been found to have an enormous negative impact on various well-being indicators including mental health [8–13]. For instance, fear associated with COVID-19 propels people to be anxious in protecting themselves and their loved ones, which can lead to social isolation, fear, and panic [14]. A recent study by Oti-Boadi et al. [11] found that Ghanaian students experienced normal to mild levels of psychological distress but reported above average scores on fear of coronavirus. In addition, a longitudinal study among youth in the United Kingdom showed an increase in mental health challenges (including increased use of maladaptive coping strategies and decreased physical activity) during the COVID-19 outbreak, especially among females and those with preexisting conditions, among other findings [13].

### Social media use during the pandemic

Almost all countries around the globe introduced social restrictive measures including quarantine and social distancing to stall the spread of the disease [15]. These measures, largely affected economic, social, and physical activities and further had a negative toll on the social, emotional, and physical well-being of people all over the world [14, 16]. The restrictive measures prevented residents from going outside of their homes and engaging in interpersonal activities and communication [14]. A significant number of the world’s population, including students were limited to activities they could do socially, thus limited to what they could do in their homes [17]. Just as mainstream media and alternative media (e.g., social media) contribute significantly to information flow about all issues and crises, the media was key in keeping people informed about the pandemic right from the outbreak. The media consistently provided information on the virus, its spread, measures of government, recommendations of the public health agencies, or the economic and consequences of the various activities around the globe [18, 19]. Social media platforms (i.e., WhatsApp, Facebook, Twitter, Instagram), in particular, proved vital to sharing information on the virus and its transmission, people’s experiences, and all other issues related to the COVID-19 pandemic [19, 20]. Similarly in Ghana, the 83.9% and 71% people described as active WhatsApp and Facebook users as of the third quarter of 2020, respectively, certainly relied on these platforms for their information about the virus [21, 22].

### Health impact of social media use

Research has established a relationship between active or high social media use and psychological well-being, as it has been found to reduce loneliness, increase life satisfaction, and provide a sense of belonging to users [23–26]. On the other hand, social media could also be disastrous to the lives of people in the sense that excessive use has been linked to grave mental health issues such as depression, anxiety, and poor sleep [27–30]. According to Gao et al. [31], the panic being spread about COVID-19 through sensational stories and misinformation by social media and other digital platforms potentially increased stress and anxiety in people. Generally, studies are showing that information received from social media were likely to cause distress in a significant proportion of the global population [20, 28, 29, 31]. Students form a greater part of the population that rely on social media for information, and this was greatly increased during the peak of the pandemic [32].

### Online-learning and students’ social media use

The COVID-19 pandemic also gravely affected the education sector globally where students had to stay at home due to closures of various educational institutions. Students mostly relied on online classes to continue their academic work and served as a basic means of maintaining the social connection between students and their colleagues [33]. Globally, the introduction of online learning and teaching stimulated the use of internet in virtually all aspects of students’ lives including surfing social media platforms for information on the pandemic [34]. Studies are showing that students online learning and adhering to stay home measures got them stressed out due to the uncertain nature of their studies and exams [31, 35]. This panicky situation strengthened their reliance on social media for communication including their learning and instructional activities [15] and to also deal with the stress and anxiety associated with the quarantine [36]. Although internet connectivity and the use of social media is limited in Low and Lower-middle income countries, research is showing that students relied on the internet and the various social platforms for their academic work, learning about COVID-19, and interpersonal communication [37]. Some studies in high-income-countries are showing that social media use has contributed significantly to the fear of contracting COVID-19 [20, 38], however, much examination of this pattern has not been done in low-income countries. Existing research, however, is showing psychological distress among students in recent times to be associated with fear of COVID-19 [11] but none have examined yet a relationship between social media use and fear of COVID-19 among students.

### Social media, gender and fear of COVID-19

The excessive use of social media during the pandemic has been linked with psychological problems. For instance, Majeed et al. [29] found that increased social media use during the current pandemic was linked to fear of COVID-19 and depression among employees. The situation was not different among students, as increased usage of social media has severe consequences for students’ psychological health [24, 39]. In addition, while reports show associations between higher to excessive use of social media and different behavioral outcomes, there may be differential impacts by gender [39–42]. For example, Xue et al. [39] found that male university students used more Social Networking Sites (SNS) and had a higher situational humor response than female university students, while female university students reported a higher fear related to COVID-19 disease. Heffner et al. [43] also found that variables such as trait anxiety, gender, and social media consumption were the strongest predictors of increasing emotional distress. Further, Hou and colleagues [44] noted in their study that while females experienced more severe stress and anxiety symptoms, males showed better resilience to stress. Females also spent more time on social media for COVID-19 related information and experienced more stress. Overall, females report more mental health issues [40], greater perceived stress [41], and showed an increase in psychiatric symptoms following the transmission of COVID-19 [42]. While highlighting the gender difference in social media usage and psychological distress, Wheaton and colleagues [20] found in their study that people experienced stressful emotions vicariously as well, where people are able to adopt the emotional reaction of others through social media.

## The current study

**Fig. 1:**
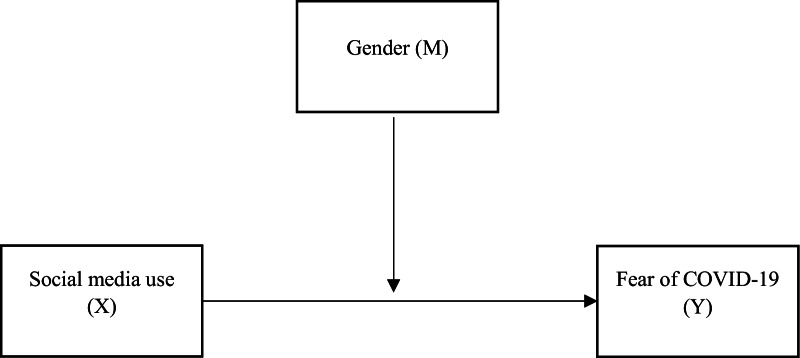
Proposed model testing the moderation of gender between social media use and fear of COVID-19

While the COVID-19 pandemic has profoundly affected people and countries physically, socially, and psychologically, the effects on student life are equally disturbing [19, 40] as many students have had to stay connected to their academic work, friends, and family through social media. Several studies have also noted that excessive use of social media during the COVID-19 pandemic contributed significantly to mental health issues experienced by students as they were exposed to myriad of information and feared stimuli presented on COVID-19 (32). During the pandemic, males used blogs, media-sharing sites, social questioning and answering and user reviews more frequently than females [45, 46], however, females were reported to have more emotional problems associated with social media use than males [24, 41]. It is therefore inevitable to ignore the gender differences in the use of social media and its potential impact on fear of COVID-19. So far, little is known about the role of gender in understanding the associations between social media and fear of COVID-19. Further, there is a paucity of research on social media and fear of COVID-19 among university students, especially in lower middle-income countries where social media usage is on the surge due to the onset of the COVID-19 pandemic. We therefore examined the influence of gender in the relationship between social media use and fear of COVID-19 amongst Ghanaian students (as shown in Fig. 1). The specific aims are as follows:

1. To examine the relationship between social media use and fear of COVID-19.
2. To examine gender differences in social media use and fear of COVID-19.
3. To examine the moderating role of gender as it relates to social media use and fear of COVID-19.

This study has the potential to contribute to better intervention programs for university students and students in general. It would also contribute to the global understanding of the impact of COVID-19 specifically, and social media broadly on mental health issues.

## Methods

### Design

This study was part of a larger project investigating the impact of COVID-19 on Ghanaian students. The study used a correlational design, collecting data exclusively online from university undergraduate students through Google Forms. The broad survey had a battery of measures assessing students’ fear of COVID-19, their coping strategies, social media usage and psychological distress. Socio-demographic characteristics were also measured.

### Participants

A total of 214 students completed the online survey but data of five students were screened out because of 50% incomplete or missing responses. Thus, a final sample of 209 participant data was examined in this study. Sample size adequacy was checked using G*Power 3.1.9.2 [47]. In G*Power, a priori power analysis was conducted based on Cohen’s [48] acceptable power of 0.80, an alpha of 0.05, a medium effect size of 0.15, and the number of predictors set at 3. Power analysis results indicated a minimum of 77 participants as sufficient to detect a medium effect. The inclusion criteria to participate in the study were (i) to be aged 18 years or older (ii) be a Ghanaian attending the University of Ghana, and (iii) understand English.

### Sampling procedure

Data from a purposive sample was collected online during the COVID-19 pandemic, from June to July 2020, among University of Ghana students via WhatsApp. Once the google form survey was created, two of the co-authors actively shared the survey link onto students’ WhatsApp individual and group platforms. On each of the two group platforms that the link was shared on, there were about 150 students. Students who voluntarily opted to participate connected to the survey by clicking on the link, provided informed consent before responding to the battery of measures. It took approximately 30 min for a participant to complete the survey. We anticipate that the survey link reached about 400 participants and therefore estimate a 54% response rate (214/400) and 52% (209/400) completion rate. Though our rates may be regarded as below the standard of 60% [49], we ensured data accuracy by actively sharing to students meeting the inclusion criteria, limiting the survey to a single entry to discourage the submission of multiple responses and using simple language to communicate instructions.

### Measures

#### Social media use integration scale [50]

This is a 10-item scale that measures social media use, emotional and behavioral responses to social cues. An example of the questions is “I post social networking updates that prompt friends to ask me what is going on.”. The scale is measured on a six-point Likert scale from 1 (strongly disagree) to 6 (strongly agree), with higher scores indicating high social media usage. This scale has been used repeatedly and has documented high reliability and validity across different samples [51, 52]. For instance, Maree [53] reported an excellent reliability coefficient of α = 0.90 and high validity values on a sample of South African students. In our study, we recorded a Cronbach’s alpha of 0.79.

#### Fear of COVID-19 [54]

The seven-item scale is a popular and well used scale that examines participants’ fear of the COVID-19 virus. A sample question is “When watching news stories about coronavirus-19 on social media, I become nervous or anxious.” Items are scored on a 5-point Likert scale with 1 = strongly disagree to 5 = strongly agree, where higher scores indicate increased fear of COVID-19. In addition to the string of data supporting the validity and reliability of this scale [11, 54–56], the Cronbach alpha for this scale within this sample was 0.86.

#### Demographic questions

Participants self-reported their actual age, gender (i.e., male or female), year in university (i.e., Level 100 to 400), type of social media apps/accounts they have and religious status (i.e., religious, or not religious).

### Ethical consideration

The study was approved by the ethics committee, Department of Psychology, University of Ghana (DREC/016/19-20). During the online data collection process, the researchers ensured strict adherence to all the necessary ethical principles of informed consent, voluntarily participation, anonymity, and confidentiality. On the information sheet, participants were additionally informed of their rights to withdraw from the research at any given time with no consequence. Participants were neither induced nor reimbursed for their participation.

### Data analysis

Data analyses were conducted in IBM SPSS version 24 with an alpha level pegged at 5% (α = 0.005). To begin, missing responses and data from five participants were dropped because of 50% incomplete or missing responses. There were no records of missing responses on any of the main study variables (i.e., gender, fear of COVID-19 and social media) and demographic variables except for ‘age’ which had six (6) missing responses, making less than 5% of the total sample size. We then performed descriptive analyses, calculating the frequencies, means, standard deviations, kurtosis, skewness and reliabilities (with Cronbach’s alpha) of the study variables. Next, an independent t-test was performed to compare gender differences on the scores of social media use and fear of COVID-19. We then examined the relationships between gender, social media use and fear of COVID-19 using Pearson’s correlation analysis. Lastly, moderation analysis (Fig. 1) was conducted with the PROCESS Macro installed over SPSS (Model 1, [57] to test the moderating effects of gender (M) between social media use (X) and fear of COVID-19 (Y). The statistical significance of the moderation was set at 95% confidence intervals (CI) with the recommended 5000 bootstrap samples [58]. Graphical tools were also used to plot the simple slope for the moderation results.

## Results

### Demographic characteristics of study participants

**Fig. 2:**
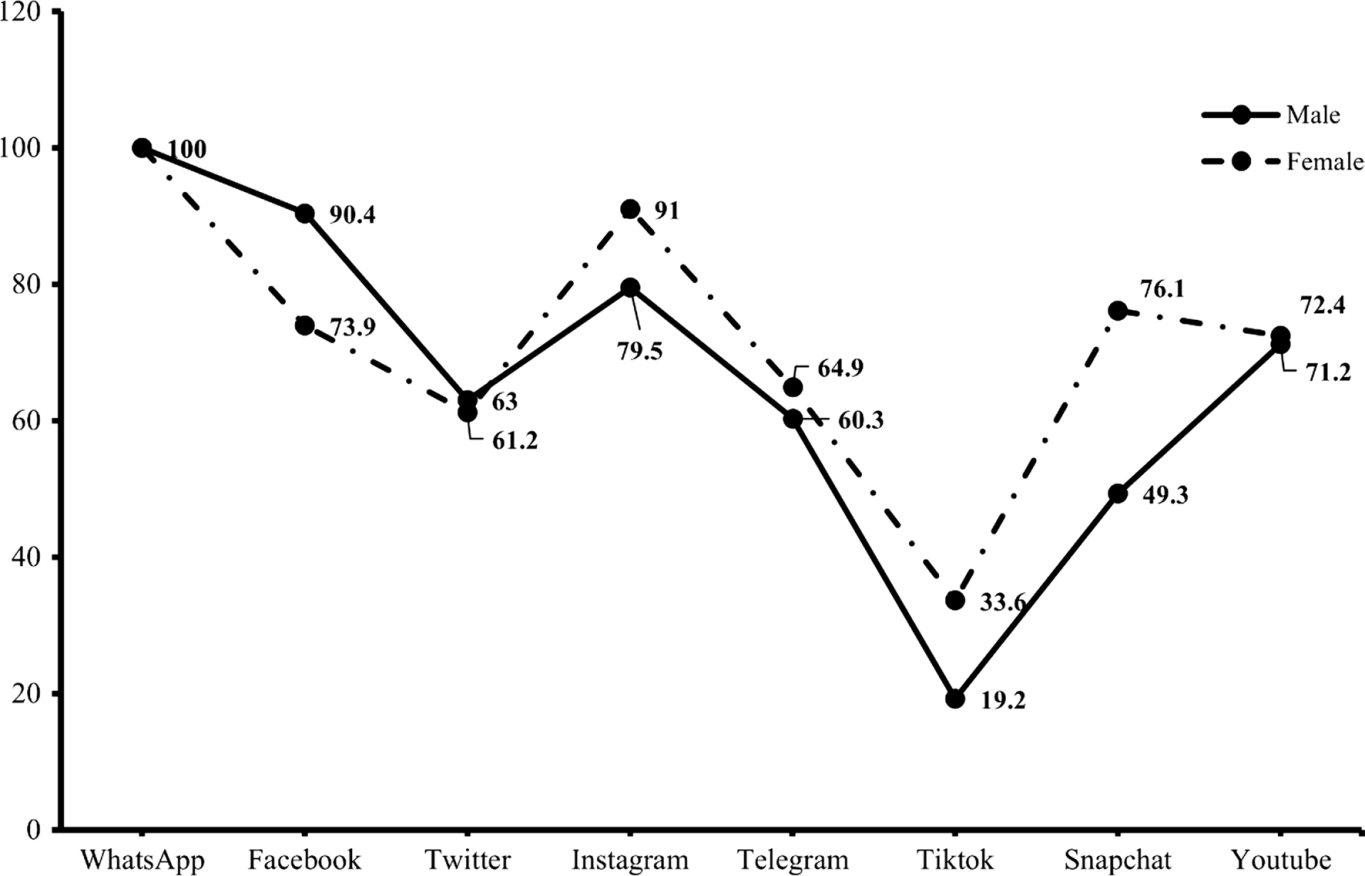
Distribution of social media applications ownership and usage by gender

Complete data for 209 participants were retained for the main analysis. The majority of the remaining 209 samples were females (64.1%), third-year students (36.8%), and were religious (93.8%). The ages of these students ranged from 18 to 28 (*M* = 21.54, *SD* = 2.04). All students were active users of social media. However, a greater proportion of students (27%) registered accounts on seven (7) different social media applications with WhatsApp being the most popular amongst males and females. In addition, more males were registered users of Facebook and Twitter whereas a female dominance was observed on the other social media applications as depicted in Fig. 2.

### Assumptions of statistical tests

Results of normality checks showed acceptable ranges of skewness and kurtosis (see Table 1). The results of reliability analysis were also within satisfactory ranges (α > 0.70). The check for multicollinearity was determined with the correlation coefficients between variables and the results from Table 1 revealed that none was highly correlated (r > 0.90). Similarly, tolerance (> 0.10) and variance inflated factor (VIF < 10) values indicated no instance of multicollinearity. The tolerance (0.89) and VIF values (1.11) of fear of COVID-19, for example, were both below acceptable cut-offs. To ensure that the data did not have any extreme outliers, the Mahalanobis distance (value 0.15), Cook's distance (value 1), and centered leverage values (value 1) were calculated. These values are far below the cutoffs. The Durbin-Watson value was 1.78, indicating that there was no residual issue. Overall, the assumptions were met in accordance with Field’s [59] recommendations.

**Table 1:** Summary of descriptive statistics, reliabilities, and zero-order correlation between study variables

Variables	1	2	3	M (SD)	α	Skewness	Kurtosis
1. Social media use	–			40.05 (7.87)	0.79	− 0.13	0.56
2. Fear of COVID-19	0.18**	–		19.45 (6.04)	0.86	0.08	− 0.28
3. Gender	0.05	− 0.013	–				

*M* = Mean; *SD* = standard deviation; α = Cronbach’s alpha

**p ≤ 0.01

### Correlational analyses and mean comparison

The only significant association recorded in the correlation analyses was between social media use and fear of COVID-19 such that there was a small and positive relationship between the variables (*r* = 0.18, *p* = 0.009). This positive relationship suggests that increasing use of social media resulted in greater experience of fear of COVID-19. See result on Table 1.

**Fig. 3:**
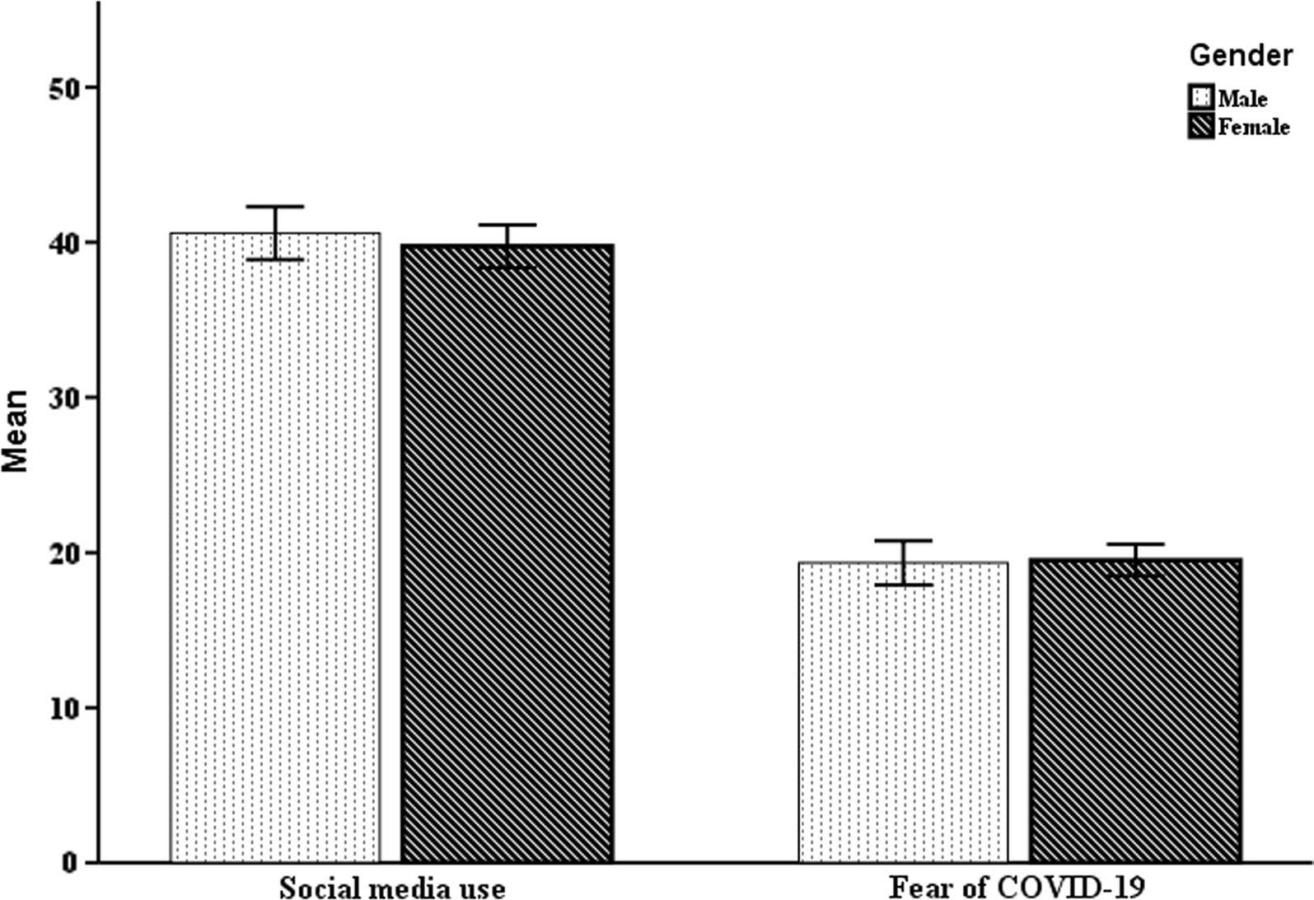
Gender differences in scores of the fear of COVID-19 and social media use

For group comparison, results as depicted in Fig. 3 showed no statistically significant gender difference, *t* (207) =  − 0.75, *p* = 0.455, in the scores of social media use, although descriptively, males (*M* = 40.59, *SD* = 7.40) scored slightly higher than females (*M* = 39.74, *SD* = 8.13). With regards to fear of COVID-19, the scores for females (*M* = 19.51, *SD* = 5.99) was marginally higher than that of males (*M* = 19.35, *SD* = 6.17), but again, this difference was not statistically significant, *t* (207) = 0.188, *p* = 0.851.

### Gender as a moderator between social media use and fear of COVID-19

**Fig. 4:**
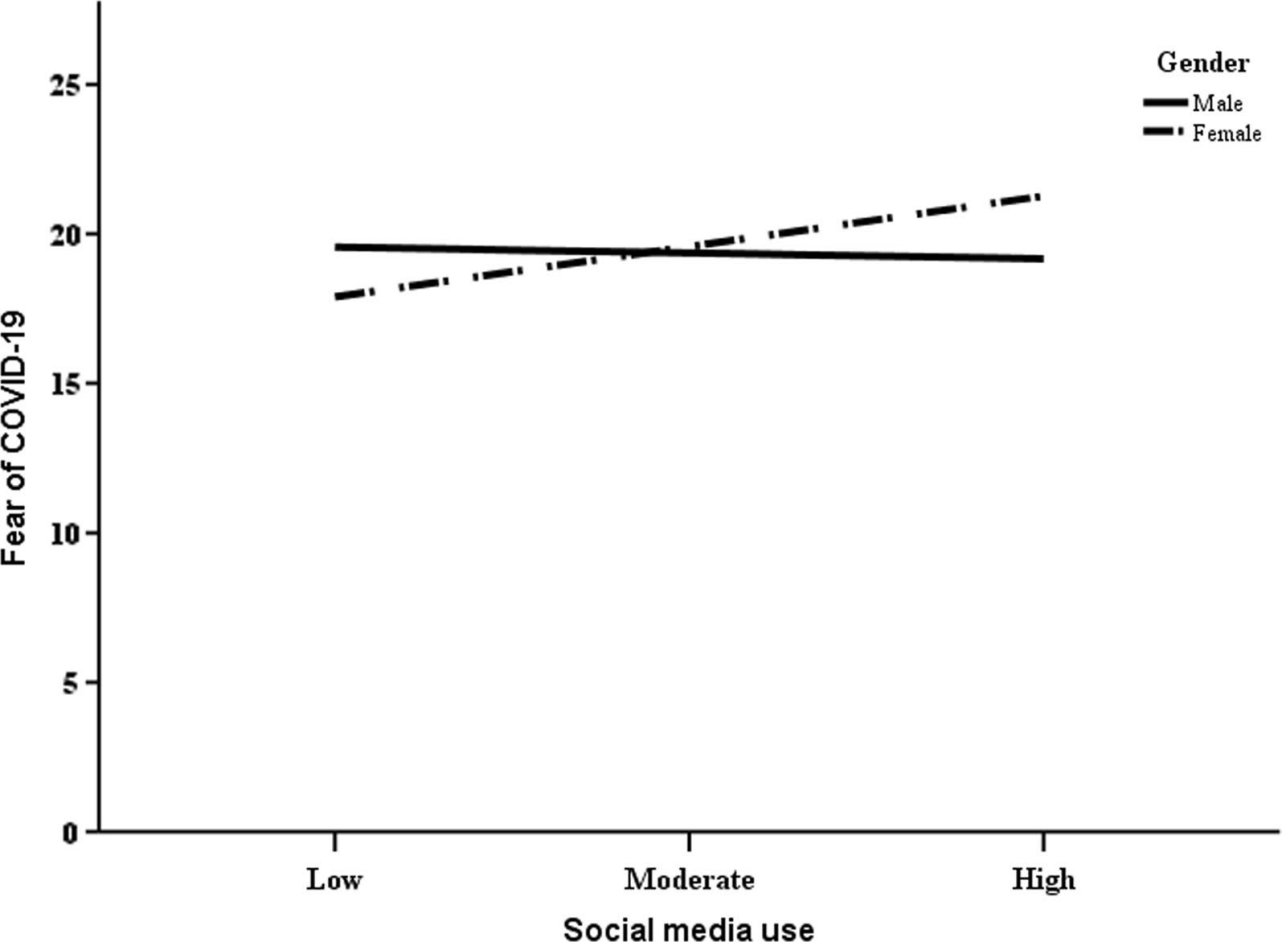
Simple slope depicting the effect of gender on the relationship between social media use and the fear of COVID-19

The PROCESS macro moderation analysis displays results similar to multiple regression models. In Table 2, the results showed that social media use was a significant predictor of fear of COVID-19 (*B* = 0.13, *p* = 0.015) but gender was not (*B* =  − 0.25, *p* = 0.801). The moderator variable was created by interacting gender and social media use. Moderation analysis showed that gender was found to be a significant moderator of the relationship between social media use and fear of COVID-19 (*B* =  − 0.24, *p* = 0.034, ∆*R*^2^ = 0.021). This significant moderation of gender added an extra 2.1% to the variance in fear of COVID-19. An examination of the interaction effect using simple slope analysis (Fig. 4) shows that while females with high usage of social media turn to report higher fear of COVID-19 (*B* = 0.22, *p* = 0.001, 95% CI: 0.90, 0.34), this interaction effect was not significant for males.

**Table 2:** Moderating effects of gender on the relationship between social media use and fear of COVID-19

	*B* (SE. *B*)	*t*	*p*	LLCI	ULCI
Constant	19.50 (0.41)	47.57	0.0000	18.69	20.30
Social media use (SMU)	0.13 (0.05)	2.46	0.015	0.03	0.23
Gender	− 0.22 (0.86)	− 0.25	0.801	− 1.90	1.47
SMU × gender	− 0.24 (0.11)	− 2.13	0.034	− 0.46	− 0.02
Model summary	*F* (3, 205) = 3.89, *p* = 0.01, *R*^2^ = 0.054				

*B* = unstandardized coefficient; *SE. B* = standard error; t = t-test score; *p* = *p* value; LLCI = lower-level confidence interval; ULCI = upper-level confidence interval; F = F-test

## Discussion

The relationship between social media use and fear of COVID-19 amongst Ghanaian undergraduate students was tested in this study. We found a small but positive relationship between these variables. No significant gender differences were observed in the mean score of these variables. Additional results from moderation analysis suggest that females with higher use of social media were more susceptible to experiencing greater fears of COVID-19.

Based on the objectives of this study, our first finding suggests that increasing use of social media was related to high rate of fear of COVID-19 amongst students. This finding corroborates with a wide range of studies that have reported the positive links of problematic social media usage with negative mental health outcomes during the early days of the pandemic, including increasing fear of COVID-19 [11, 29, 38], perceived threat and panic of COVID-19 [24, 60], emotional loneliness [9, 61], psychological distress, anxiety, and depression [10, 43, 62]. Since the COVID-19 outbreak, and due to limited movements and social restrictions, there has been a record surge in the use of social media among the youth to gather and share information about the pandemic, communicate with loved ones, to study and finish assignments, among others [63]. Despite the many usefulness of social media platforms, the same also served as a repository for continuous local and global information related to the number of people contracting the virus, the number of people in isolation centers, the death rates in various countries, misinformation and misconceptions about the virus and care of patients [64, 65]. A mix of accurate and inaccurate information amid the global uncertainty was rife during the data collection period. One example of misinformation that was widespread in social media among Ghanaians was that COVID-19 was being used as a biological weapon against developing economies [22]. Since negative and fear-laden news generally spreads faster than healthy positive news, such negative pieces of information and news get more attention on social media about COVID-19, and thus had the potential of creating fear and panic amongst people including our sample [6].

Regarding gender, although no significant gender differences in social media use and fear of COVID-19 were found, our results from moderation analysis suggest that females who reported higher use of social media were inclined to experience greater fear of the pandemic. This is consistent with studies by Broche-Pérez et al. [46] and Alsharaway et al. [45] which revealed higher levels of COVID-19 fear and risk perception among females. For example, in a related study that focused on younger school students than our sample, Radwan and colleagues [24] found that females consumed a higher amount of COVID-19 related news than males from Facebook. According to Alsharaway et al. [45], females in their study were likely to experience COVID-19 fear more than males because of higher affective intensity, making females more emotionally predisposed to experiencing and expressing negative emotions. Bearing this in mind, females in our study who maintained prolonged social media usage during the peak of the pandemic may have been vulnerable to consuming negative COVID-19 related information and this may have caused emotional distress and heightened fear. The gender differences in fear could also be explained with the gender role socialization theory [66]. According to this theory, the expression of fear is incompatible with the masculine gender role in many cultures including the Ghanaian culture; thus, males are encouraged to suppress or minimize their fears. In contrast, the feminine gender role encourages girls to express their distress and seek social support rather than muting their fears.

### Implications of study findings

The findings of this study call on stakeholders and social media content creators to implement programs that educate and raise public awareness on the appropriate use of social media and ways to identify false spread of information including those related to COVID-19. Also, stakeholders should invest in establishing new or augmenting existing youth-friendly online mental health services and interventions to assist individuals who have fears or anxiety because of negative news about COVID-19 and future issues displayed on social media. Finally, tutorials on critical thinking and self-care may be relevant to guide students to identify stressors from social media and the environment, as well as simple self-care strategies. This would be useful in the era of limited-in person contact from counsellors and health professionals. Currently as we write (May 2021), COVID-19 still exists predominantly as the OMICRON variant [67]. We cannot be certain whether this would be the last variant, therefore interventions mentioned are still relevant for the times and in preparation for future outbreaks.

### Strengths and weaknesses

Even though our study employed a large sample size and data was collected using rigorous procedures, it does have some limitations worth mentioning to guide future research. First, data were gathered via an online self-report questionnaire. It is possible that participants’ bias and intentional misreporting will have an impact on data accuracy. Furthermore, the study only looked at the relationship between social media and the fear of COVID-19, and not whether social media had an impact on students’ fear and anxiety levels before the pandemic. Future studies could examine retrospectively pre-COVID 19 fear and anxiety levels and compare to levels during the COVID-19 era. Finally, because the dataset was gathered in a cross-sectional fashion, we only report correlational findings rather than cause-and-effect relationships. It is suggested that future research explore these variables using a longitudinal design.

## Conclusion

The relationship between social media use and fear of COVID-19 amongst Ghanaian undergraduate students was examined. We found out that social media use had a small and positive correlation with fear of COVID-19. Findings also revealed that excessive usage of social media and the heightened levels of fear of COVID-19 was significant only among females. It is therefore critical to consider educating youth on how to and encouraging them to best utilize media platforms to help overcome some of the psychological issues associated with COVID-19. Additionally, health professionals and educational personnel including school counsellors and psychologists can use the information provided in this study to design more gender-specific interventions to support students’ wellbeing, especially females.

## Data Availability

The dataset generated and/or analyzed during the current study is available from the corresponding author on reasonable request.
